# Totally Percutaneous Access Using Perclose Proglide for Endovascular Treatment of Aortic Diseases

**DOI:** 10.21470/1678-9741-2016-0065

**Published:** 2017

**Authors:** Eduardo Keller Saadi, Marina Saadi, Rodrigo Saadi, Ana Paula Tagliari, Bernardo Mastella

**Affiliations:** 1 Hospital de Clínicas de Porto Alegre (HCPA), Universidade Federal do Rio Grande do Sul (UFRGS), Porto Alegre, RS, Brazil.; 2 Universidade Luterana do Brasil (ULBRA), Canoas, RS, Brazil.; 3 Pontifícia Universidade Católica do Rio Grande do Sul (PUCRS), Porto Alegre, RS, Brazil.; 4 Universidade Federal do Rio Grande do Sul (UFRGS), Porto Alegre, RS, Brazil.

**Keywords:** Aortic Diseases, Aortic Aneurysm, Endovascular Procedures, Femoral Artery, Suture Techniques/Instrumentation

## Abstract

**Objective:**

To evaluate our experience following the introduction of a percutaneous program for endovascular treatment of aortic diseases using Perclose Proglide® assessing efficacy, complications and identification of potential risk factors that could predict failure or major access site complications.

**Methods:**

A retrospective cohort study during a two-year period was performed. All the patients submitted to totally percutaneous endovascular repair (PEVAR) of aortic diseases and transcatheter aortic valve implantation since we started the total percutaneous approach with the preclosure technique from November 2013 to December 2015 were included in the study. The primary endpoint was major ipsilateral access complication, defined according to PEVAR trial.

**Results:**

In a cohort of 123 patients, immediate technical success was obtained in 121 (98.37%) patients, with only two (0.82%) cases in 242 vascular access sites that required intervention immediately after the procedure. Pairwise comparisons revealed increased major access complication among patients with >50% common femoral artery (CFA) calcification *vs.* none (*P*=0.004) and > 50% CFA calcification *vs.* < 50% CFA calcification (*P*=0.002). Small artery diameter (<6.5 mm) also increased major access complication compared to bigger diameters (> 6.5 mm) (*P*=0.027).

**Conclusion:**

The preclosure technique with two Perclose Proglide® for PEVAR is safe and effective. Complications occur more often in patients with unfavorable access site anatomy and the success rate can be improved with proper patient selection.

**Table:** 

Abbreviations, acronyms & symbols	
ACT CFA CEVAR EVAR ICU PEVAR SD SEVAR SPSS TAVI TEVAR	= Activated clotting time = Common femoral artery = Common femoral artery endovascular repair = Endovascular aneurysm repair = Intensive care unit = Percutaneous endovascular repair = Standard deviation = Surgical endovascular repair = Statistical Package for Social Sciences = Transcatheter aortic valve implantation = Thoracic endovascular aortic repair

## INTRODUCTION

Endovascular treatment has been the first option of many thoracic and abdominal aortic diseases, as well as transcatheter aortic valve implantation (TAVI) in the last years. The access is usually the surgical exposure of the common femoral arteries (CFA) to introduce the delivery system.

Arteriotomy closure devices were introduced in 1995 to decrease vascular complications and reduce the time to hemostasis and ambulation. Subsequently, several generations of passive and active arteriotomy closure devices have been introduced that incorporate suture, collagen plug, nitinol clip and other mechanisms to achieve hemostasis^[1]^. In 1999 there was the advent of preclosure technique advocated by Haas et al.^[2]^.

Subsequently, the preclosure technique was proven to be safe for bigger sheaths and then, the totally percutaneous endovascular repair (PEVAR) approach from the femoral arteries has been applied as an alternative to surgical cutdown^[3]^. Even being less invasive than surgical cutdown at the vascular access, the use of total percutaneous approach has not gained broad acceptance.

Few studies have reported the results and our aim is to verify the safety and efficacy of this technique in a clinical series.

## METHODS

Registry of consecutive patients submitted to percutaneous endovascular procedures to treat aortic diseases identified from our prospectively maintained database. This clinical observational study was approved by the research and ethics committee of our institution, under the number 16-0124.

All the patients submitted to complete percutaneous treatment for aortic diseases and TAVI since we started the total percutaneous approach with the preclosure technique from November 2013 to December 2015 were included in the study that analyzed major and minor access site complications and correlate with some potential risk factors.

A major ipsilateral access complication was defined according to PEVAR trial: 1) an access site vascular injury requiring repair, 2) new onset of lower-extremity ischemia necessitating surgical or percutaneous intervention, 3) access site-related bleeding necessitating transfusion, 4) access site-related infection necessitating antibiotics, drainage or prolonged hospitalization and 5) acute pseudoaneurysm.

A secondary endpoint of minor ipsilateral access site vascular sequelae was also evaluated. Minor sequelae included 1) pseudoaneurysm/arteriovenous fistula, 2) hematomas > 6 cm, 3) post-discharge bleeding necessitating > 30 minutes to reach hemostasis, and 4) deep venous thrombosis. Another secondary endpoint evaluated was late follow-up by angiotomography in the 3^rd^ month and then yearly as arterial thrombosis, arterial dissection, pseudoaneurysm, stenosis > 30%, arteriovenous fistula and hematoma. Any percutaneous accesses with introducer ≥ 12 F were analyzed independently as access site.

The exclusion criteria for the study was incomplete data collection or lost follow-up. There was just one patient excluded from the study that died after TAVI due to acute coronary occlusion and all other consecutive patients in that period were included.

### Technique for Vascular Closure Device Implantation

All the procedures were performed by the same operator with the use of the Perclose Proglide® (Abbott Vascular, CA, USA) device. Previous training in a simulator and 50 procedures were performed delivering a single perclose in sheaths less than 9 F. Only then, the use of the preclose technique was started for bigger introducer systems.

The procedures were performed under general, regional or local anesthesia, depending on the patient evaluation by the surgeon and anesthetist. The puncture site was evaluated by preoperative angiotomography, in order to identify the bifurcation level, the size, tortuosity and the grade of calcification of the CFA and external iliac arteries. After a small incision in the skin (2 mm) with an 11 blade, the CFA was punctioned with an 18 needle using the Seldinger technique, avoiding the posterior wall. No ultrasound was used to guide the puncture. A short 6 F introducer was inserted first in the right CFA. A sharp dilatation around the sheath with the dilator inside was performed with a Kelly in the subcutaneous tissue, in order to facilitate the knots tying at the end of the procedure. Two Percloses Proglide® were then inserted through a short 0.035 wire, starting from the surgeon’s side (right) to the left of the patient. The first Perclose Proglide® was positioned at 11 o’clock and the second one at 1 o’clock. The 3-0 prolene of the device was gently repaired in moist gauze without tension. After the insertion of the second vascular closure device, a 7 F short sheath was inserted to avoid bleeding. The same step was done in the left femoral artery. At this time, heparin was given at 1 mg/kg to keep the activated clotting time (ACT) around 250 seconds.

The endovascular procedure was carried out as usual with the bigger sheaths insertion and endoprosthesis implantation. At the end of the procedure, the assistant compressed manually the puncture site as the surgeon removed the introducer, leaving a hydrophilic wire in place, while the knots were tied, starting from the right towards the left. The first knot (right) approximates the artery pulling the blue suture thread. If there was no pulsatile bleeding, the wire was removed and the knot pushed to the artery with the trimmer, tied with the white thread and cut. The second knot (left) was then tied. The same step was done in the left femoral artery, starting from the right side of the patient. Heparin was reversed half dose and manual compression was maintained for at least 5 minutes. If any bleeding was noted, further compression was applied.

### Success Definition and Follow-Up by Angiotomography

Immediate success was defined as good vascular closure device (Perclose Proglide®) delivery with adequate knot tightening and closure of the CFA without any bleeding, vascular occlusion, open conversion or further endovascular intervention for artery repair in the first 30 days after the procedure. Blood transfusion, groin hematoma or lymphocele were also evaluated.

All the patients were followed up clinically and with an angiotomography of the aortic correction and the vascular access site during three months and then, yearly to evaluate late access-related complications. The images were analyzed independently and separately by a radiologist and a surgeon. Complications considered at the access site by angiotomography were: arterial thrombosis, dissection, pseudoaneurysm, stenosis > 30% and arteriovenous fistula. The absence of any of these complications was considered late success.

### Statistical Analysis

The software Statistical Package for Social Sciences (SPSS) 18.0 was used for data processing and statistical analysis.

Continuous data were presented as mean ± standard deviation (SD). Categorical data were described as number and percentages. Student’s t-test was used to compare continuous data and Chi-square test to compare categorical data in order to evaluate success and complications. Binary logistic regression and one-way ANOVA analysis were used to detect outcome predictors. A *P* value <0.05 was considered statistically significant.

## RESULTS

Demographic and procedure characteristics are presented in Tables 1 to 3, respectively. Immediate technical success was obtained in 121 (98.37%) patients, with only 2 (0.82%) cases in 242 vascular access sites that required intervention immediately after the procedure (Table 4). In both patients, severe ischemia was detected by physical examination in the hybrid room and prompt surgical approach of the femoral arteries. One patient had thoracic endovascular aortic repair (TEVAR) with a 20 F sheath and the other an endovascular aneurysm repair (EVAR) with an 18 F introducer. They were female, with small (< 6.5 mm) and calcified (> 50%) access vessels. During the surgical exposure, a plaque obstruction was observed. In one case, a resection with a direct end-to-end anastomosis was done in the CFA and, in the other patient, a 7 mm polytetrafluoroethylene graft was interposed between external iliac and CFA. Both patients had a good and uneventful postoperative course.

**Table 1:** Demographic characteristic.

Variables	n (%)	Mean (interval)
Patient	123 (100)	__
Age (years)		76.80 (43 – 95)
Sex		
Male	76 (61.8)	__
Female	47 (38.2)	__
Comorbidities		
Hypertension	95 (77.2)	__
Diabetes	39 (31.7)	__

**Table 3:** Procedure characteristic – specified by the procedure side.

Variables	Left side		Right side	
	n (%)	Mean (interval)	n (%)	Mean (interval)
CFA minimum diameter (mm)	__	7.8 (5 – 10)	__	7.8 (4 – 10)
CFA calcification				
0	37 (30.1)	__	46 (37.4)	__
≤ 50%	72 (58.5)	__	59 (48)	__
> 50%	14 (11.4)	__	18 (14.6)	__
Number of access sites ≥ 12 F	60 (48.8)	__	112 (91.1)	__
Sheath size	__	11.2 (6 – 24)	__	17.0 (6 – 26)
< 18 F	89 (72.4)	__	23 (18.7)	__
≥ 18 F	34 (27.6)	__	99 (80.5)	__

CFA=common femoral artery

**Table 4:** Acute major vascular access complication.

Complications	n (%)
Bleeding that needed vascular intervention or transfusion	__
Limb ischemia - Acute arterial dissection/occlusion	2 (1.62%)
Acute pseudoaneurysm	__
Infection	__

There were no cases of vessel puncture site hematoma and only one patient required a perioperative blood transfusion, which was unrelated to the access site (Table 4).

One patient presented a minor late complication (Table 5) detected on a routine angiotomography in the postoperative period (three months after the procedure). It was a small (0.4 cm) asymptomatic pseudoaneurysm at the puncture site in the right CFA. The patient is being followed with Doppler ultrasound and angiotomography, with no symptoms or increase in size up to a 18-month follow-up period. This was a TEVAR with a 20 F sheath in a 72 year old woman with good sizes (7.5 mm) and not very calcified (< 50%) access vessels.

**Table 5:** Late complications after 30 days - angiotomography

Complications	n (%)
Arterial thrombosis	__
Arterial dissection	__
Pseudoaneurysm	1 (0.81%)
Stenosis > 30%	__
Arteriovenous fistula	__
Hematoma	__

In three patients the Perclose Proglide® could not be delivered at the first attempt, since it did not crossed the anterior wall. In these situations, we simply removed the device and inserted another one over the short wire, with success at the second attempt in all cases.

Pairwise comparisons revealed increased major access complication among patients with >50% CFA calcification *vs.* none (*P*=0.004) and > 50% CFA calcification *vs.* < 50% CFA calcification (*P*=0.002). Small artery diameter (<6.5 mm) also increased major access complication compared to bigger diameters (>6.5 mm) (*P*=0.027). Other possible predictors of major access complications were analyzed and can be seen in Table 6.

**Table 6:** Risk factors for major vascular access complications. Univariate analysis.

Characteristic	Major complication	*P* value
Age (complication *vs*. no complication)	76.63 ±9.6 *vs*. 84±8.5 years	0.619
Female *vs*. male gender	1/47 (2.12%) *vs*. 0/76	0.816
CFA calcification		0.005
0	0/33	
<50%	0/70	
>50%	2/20 (10%)	
CFA diameter (mm)		0.027
<6.5	2/21 (9.5%)	
>6.5	0/102	
Introducer		1
<18 F	0/15	
≥18 F	2/108 (1.9%)	

CFA=common femoral artery

## DISCUSSION

The first multicenter randomized controlled trial published by Nelson et al.^[3]^ included 151 patients undergoing EVAR using either the Prostar XL or Perclose Proglide® (PEVAR) *versus* standard cutdown of the CFA endovascular repair (CEVAR). Their study demonstrated a technical success rate of 96% with the use of Perclose Proglide®, which was similar to our findings.

With a trend towards less invasive approach, a recent study comparing EVAR with PEVAR showed a technical success rate of 96% in PEVAR patients, a significantly shorter total operation time, a shorter length of hospital stay, and fewer wound complications in patients who were treated with PEVAR^[4]^.

In our study we used the same definition of access-related complications as used in the PEVAR trial. As our main goal was to evaluate the results of vascular closure device and complications with large sheaths, several aortic pathologies such as aneurysms and transcatheter aortic valve implantation were included.

A high rate of success in device deployment (98.37%) was found, as well as a small number of complications. Furthermore, these complications were more restricted to our initial learning curve (cases number 7, 11 and 35). In our series, there were two (1.61%) major vascular complications, both were acute vascular occlusion, which were successfully treated with immediate open repair (cases number 11 and 35). There was only one (0.81%) minor late complication (a small 0.4 cm asymptomatic pseudoaneurysm which has been followed for 18 months - case number 11). This corresponds to 0.82% of vascular accesses in our series with Perclose®.

Even though our objective wasn’t to compare PEVAR with Surgical Endovascular Repair (SEVAR), is worth to remember that the PEVAR trial demonstrated a significant advantage over the PEVAR treatment regarding major ipsilateral access site vascular sequelae in 30 days (6% *vs.* 10%; *P*=0.0048). However, for minor ipsilateral access site vascular sequelae, the authors found similarity between the groups (4% *vs.* 8%; *P*=0.6777).

Three (2.43%) patients could not use the device at the first attempt. The needles didn’t cross the arterial wall and when we pull to cut the first thread. We simply removed the entire device and inserted another one, with success in all three cases. This could be due to some device defect; or, more probably, to CFA calcification - wall stiffness impeding the needle to cross the arterial wall; or to fibrosis around the artery. It did not compromise percutaneous closure success. This, per se, was not considered a failure because another device was introduced and adequate hemostasis was obtained in all patients.

We also demonstrate that the percutaneous closure is safe and effective in a clinical series with a short operative time and no wound complications in PEVAR patients. Our average length of unit intensive care (ICU) stay was 24±14.25 hours and our average length of hospital stay was 3±3.1 days.

Prior published reports success rates for PEVAR varying from 71% to 100%^[4-8]^. In addition to access vessel diameter and type of closures device, femoral artery calcification, access vessel tortuosity and depth of CFA and groin scars have all been associated with PEVAR failure^[9-11]^.

Some previous studies have shown that the success rate is significantly related to the caliber of the sheath^[12-15]^. Lee et al.^[16]^ demonstrated that large sheaths had low technical success rates among the subset of sheath sizes. Kim et al.^[17]^ also found seven of eight closure procedure failures in cases involving sheaths over 18 F and believed that procedural failure might be related to large sheath sizes. Sheath size ≥ 18 F is considered as a possible predictor of percutaneous vascular complications in PEVAR, and this is why these complications occur more often in thoracic stent grafts, which usually use delivery sheaths larger than 18 F. In other review articles^[9,14]^ the CFA diameter was considered another predictor of vascular failure. Similarly to these studies, we also demonstrated that > 50% CFA calcification and < 6.5 mm CFA diameter were predictive of major complications. In our study, both patients that had acute vascular complications were female, had 18 F or bigger sheath and small (6.5 mm) and calcified (>50%) access vessels. The association of big sheath (≥18 F) and small vessels with calcification is believed to be a bad combination for percutaneous approach.

In one meta-analysis, the quality of the artery was found to be a greater predictor of failure than the sheath size itself^[12]^. The use of the Perclose Proglide® device is not considered when the CFA has a large plaque (> 50% circumference) or ring-shape calcification. Patients with > 50% anterior wall calcification had a higher failure rate than patients without calcification^[18]^.

All of our patients that had acute vascular complications (2 - 1.62%) had more than 2/3 CFA calcifications. Based on this experience, in the case of severe calcification (> than 2/3 and anterior plaque), we would prefer a CFA cutdown or an alternative access instead. It was made clear that the association of small femoral or iliac arteries (<6.5 mm), severe calcification (> than 2/3 particularly in the anterior wall) and tortuosity is bad for any femoral access, especially for PEVAR.

During midterm follow-up vascular complications such as infection, thrombosis, stenosis, occlusion or pseudoaneurysm are rare^[18]^. All of our patients were followed clinically and with an angiotomography for vascular access evaluation analyzed independently by a radiologist and a surgeon, with just one (0.81%) patient presenting with a small (4 mm) asymptomatic pseudoaneurysm that is being followed every 6 months with imaging.

The American Heart Association currently recommends the use of femoral artery closure devices to achieve faster hemostasis, shorter duration of bed rest, and possibly improved patient comfort^[1]^.

The preclosure technique is now widely used and started with a 10 F ProStar® XL (Abbott Laboratories, Abbott Park, Il, USA). More recently the use of multiple 6 F Perclose Proglide® devices has been recommended for bigger sheaths. The ProStar® XL, which was the first reported closure device for PEVAR^[2]^, has several disadvantages compared with multiple Perclose Proglide®. First, the ProStar® XL requires more extensive subcutaneous dissection to insert. Second, the mechanism of deployment is complicated, and failure of hemostasis could occur early in the experience (learning curve). Third, a braided suture may have increased the potential for infections. The Perclose Proglide® has a small profile (6 F) and a simple deployment mechanism with a monofilament (prolene 3-0) suture very similar to surgical arterial suture. We started using the Perclose Proglide® and we have no experience with the ProStar® XL.

Total percutaneous access with the use of Perclose Proglide® in our experience showed to be effective, with a very few complications in the setting of PEVAR for the treatment of several aortic and aortic valve pathologies. Recently, the use of just one Perclose Proglide®, in order to reduce costs, has been reported with very good results^[19]^.

### Limitations

This study has some limitations. It was a retrospective, nonrandomized and observational study with a relatively small number of patients at a single center. Our findings should be prospectively confirmed with a larger population.

## CONCLUSION

The preclosure technique with two Perclose Proglide® for PEVAR proved to be safe and effective. There was no mortality related to the technique in our series and two acute femoral artery complications that were more restricted to our initial learning curve and required prompt vascular intervention. Both patients were female, had small (6.5 mm) and more than 50% CFA calcification and these three situations predicted vascular access complications.

With a very meticulous technique, well-trained surgeon and careful patient selection, total percutaneous approach to endovascular treatment for several aortic diseases is safe and less invasive than CFA cutdown. The mortality and morbidity of PEVAR were low, but longer follow-up is necessary.

**Table:** 

Authors’ roles & responsibilities	
EKS	Conception and study design; realization of operations; analysis and/or data interpretation; statistical analysis; manuscript redaction or critical review of its content; final manuscript approval
MS	Conception and study design; analysis and/or data interpretation; final manuscript approval
RS	Conception and study design; analysis and/or data interpretation; final manuscript approval
APT	Conception and study design; realization of operations; analysis and/or data interpretation; statistical analysis; final manuscript approval
BM	Conception and study design; analysis and/or data interpretation; final manuscript approval

## Figures and Tables

**Table 2:** Procedure characteristic.

Variables	n (%)	Mean (interval)
Procedure type		
TEVAR	17 (13.7%)	__
TAAA	4 (3.2%)	__
EVAR	44 (33.5%)	__
TAVI	59 (47.6%)	__
Procedure time (minutes)	__	70 (20 – 190)
ICU time (hours)	__	28.5 (12 – 48)
Hospital stay (days)	__	3.8 (2 – 35)
Blood transfusion	1 (0.8)	__
CFA minimum diameter (mm)	__	7.8 (4 – 10)
CFA calcification		
0	83 (35)	__
≤ 50%	131 (55.3)	__
> 50%	32 (9.7)	__
Number of access site ≥ 12 F	172 (69.9)	__
Sheath size		
< 18 F	112 (45.7)	__
≥ 18 F	133 (54.3)	__

EVAR=endovascular repair for abdominal aortic aneurysms; TEVAR=thoracic endovascular repair; TAAA=thoracoabdominal aortic aneurysms; TAVI=transcatheter aortic valve implantation; CFA=common femoral artery; ICU: Intensive care unit
